# Xylan epitope profiling: an enhanced approach to study organ development-dependent changes in xylan structure, biosynthesis, and deposition in plant cell walls

**DOI:** 10.1186/s13068-017-0935-5

**Published:** 2017-11-30

**Authors:** Angelo G. Peralta, Sivasankari Venkatachalam, Sydney C. Stone, Sivakumar Pattathil

**Affiliations:** 1 Complex Carbohydrate Research Center, University of Georgia, 315 Riverbend Road, Athens, GA 30605 USA; 2 BioEnergy Science Center, Oak Ridge National Laboratory, Oak Ridge, TN 37831 USA; 3 Present Address: Mascoma LLC (Lallemand Inc.), 67 Etna Road, Lebanon, NH 03766 USA

**Keywords:** Glycome profiling, Xylan, Development, Epitope characterisation

## Abstract

**Background:**

Xylan is a major hemicellulosic component in the cell walls of higher plants especially in the secondary walls of vascular cells which are playing important roles in physiological processes and overall mechanical strength. Being the second most abundant cell wall polymer after cellulose, xylan is an abundant non-cellulosic carbohydrate constituent of plant biomass. Xylan structures have been demonstrated to contribute to plant biomass recalcitrance during bioenergy applications. A critical understanding of xylan composition, structure, and biosynthesis in developing plant stems will allow an increased understanding of how cell walls are put together in this organ in a basic research, and, in applied research, will improve strategies in xylan engineering to reduce biomass recalcitrance for economically feasible biofuel production.

**Methods:**

We describe an approach to enable the monitoring of xylan epitope structures in cell walls during the stem maturation process in Arabidopsis. The technique integrates glycome profiling, an in vitro immunoanalytical platform, and in situ immunolocalisation to provide comprehensive details on the presence, relative abundances, and dynamics with which diverse xylan epitope structures are integrated to the cell walls throughout the stem maturation process.

**Results:**

Our experimental results and the supporting in silico analysis demonstrated that xylan deposition in stems occurs early on in stem development; however, xylan epitope types (representing substituted and unsubstituted regions on xylan backbone made of β-(1,4)-linked xylose residues) and the strength of their integration into the final wall structure vary during stem maturation.

**Conclusions:**

Our novel approach thus provides a method to comprehensively survey the differences in xylan epitope patterning and deposition occurring in stem development and thereby providing a robust tool for characterising altered xylan integration patterns in cell walls during the stem maturation process in diverse plant cell wall biosynthetic mutants. Our findings also suggest that this approach could rapidly and reliably delineate xylan deposition patterns in the cell walls of plants belonging to diverse phylogenetic classes providing novel insights into the functional roles of xylans in overall growth and development.

**Electronic supplementary material:**

The online version of this article (doi:10.1186/s13068-017-0935-5) contains supplementary material, which is available to authorized users.

## Background

Cell walls perform a number of important roles in plant growth and development including providing shape to different cell types, act as an interface between adjacent cells, intercellular communication, and defence responses against pathogenic attack. Cell walls in mature plant cells are structurally divided into primary cell walls, which surround expanding cells and secondary cell walls that are thickened structures containing lignin in order to provide structural support to the plant [1]. The major components of plant secondary walls are cellulose, hemicellulose (that include xylan, xyloglucan, and glucomannan), and lignin [2]. One of the major hemicellulose components in secondary cell walls, xylan, is a linear cell wall polymer that consists of a backbone made of β-(1,4)-linked xylose residues that is generally substituted with arabinose, acetyl, glucuronic acid (GlcA), and 4-*O*-methylglucoronic acid residues at varying degrees depending on plant species [3]. In dicots, the type I primary cell wall predominantly consists of glucuronoxylan (GX) with a linear backbone of β-(1,4)-linked xylose (Xyl) residues that are substituted with α-(1,2)-linked glucuronic acid (GlcA). These GXs occur as either binding tightly to exposed faces of glucan chains in cellulose microfibrils, and spanning the distance between adjacent microfibrils or with other GXs to space and lock cellulose microfibrils into place [4, 5]. In contrast, type II primary walls, characteristic of monocots are composed of glucuronoarabinoxylans (GAX) which attach to cellulose microfibrils in a similar fashion to type I walls. The type I and type II primary walls differ from the secondary cell walls of dicots including *Arabidopsis*, where xylan is the major hemicellulosic polymer present in the wall where they are actively being biosynthesised [6, 7]. Along with cellulose and lignin, xylan represents one of the main structural components in xylem vessels that facilitate the rapid movement of water while maintaining its structural integrity despite the negative pressure that occurs. In interfascicular fibres, xylan contributes to the thickness of the wall in fibre cells which allows them to maintain the mechanical strength of the stem [8, 9].The importance of xylan as a major constituent of the secondary cell wall in stems is well-emphasised by xylan-deficient mutants exhibiting weakened stems [10]. Xylan has been shown to exhibit varying structural features in plant cell wall throughout the development. For example, in wheat seedlings; specifically, arabinoxylan content changed from a high degree of arabinose substitution to a much lower degree of substitution, highlighting the complex manner in which high- and low-substituted arabinoxylans are deposited in primary and secondary cell walls in different cell types and at different development stages [11]. In developing willow stems, it was observed during stem maturation that xylan content and the degree of methylation of its GlcA side chains increase. Additionally, immunolabelling of xylan distribution using LM10 monoclonal antibody demonstrated increased labelling of unsubstituted and substituted xylan epitopes as the secondary xylem developed [12].

Since xylans are the second most abundant cell wall polymer after cellulose, they are a major non-cellulosic component of plant biomass. The xylose monomer units in xylan, however, are incompatible for fermentation into biofuels and other bio-products by organisms such as yeast due to their pentose structure [3]. Xylan also has a contributing role in cell wall recalcitrance by (1) cross-linking with lignin through ester bonds to GlcA and ether bonds with Xyl or Ara and (2) the ferulic acid dimerisation in grass xylans results in cross-linking of adjacent xylan chains or to lignin [3, 13, 14]. Additionally, these ferulic acid esters negatively impact xylan and cellulose hydrolysis [3]. Understanding xylan composition, structure, and biosynthesis is of key significance in designing strategies for engineering xylan in plants with improved properties such as decreased recalcitrance for biofuel production.

Advances have been made in gaining insights into the co-expression analysis of genes directly or indirectly associated with xylan biosynthesis including putative glycosyltransferases [15]. Several recent studies have focused on xylan structural variations during primary and secondary cell wall formation in Arabidopsis (see review by Hao and Mohnen [16]); however, there are only a handful of information on the structural dynamics of xylan in primary and secondary cell walls especially as a function of developmental stages of plant organs [17]. Moreover, despite early attempts to map the spatial and temporal distribution of xylans depending on developmental stages [12, 18], there are still no comprehensive studies on development-dependent variations pertaining to xylan sub-structures during stem maturation process. Understanding the differentiation of xylan deposition in a developmental context would provide a clearer picture of spatio-temporal regulation of xylan biosynthetic process and hence overall cell wall biosynthetic process in plant organs.

Plant cell wall glycan-directed monoclonal antibodies (mAbs) are highly specific probes used in plant cell wall analyses [19]. mAbs can bind monospecifically to glycan sub-structures, also known as glycan epitopes. This monospecificity feature provides mAbs with distinct advantages: it can bind to glycans whose structures are frequently repetitive and found in multiple macromolecular contexts (for example, arabinogalactan epitopes that are present in glycoproteins and pectic polysaccharides such as rhamnogalacturonan-I) and more importantly, their epitope binding specificity can be determined unambiguously. These mAbs can be used through either (1) in vitro detection through glycome profiling and (2) in situ visualisation by immunohistochemical methods. Glycome profiling (GP) involves sequential extraction of cell wall samples using a series of reagents increasing in harshness and then ELISA-screening these isolated extracts with mAbs in order to determine and monitor the glycan epitopes released by each extraction. Sequential extractions allow cell wall glycans to be isolated in extracts on the basis of the relative tightness with which they are integrated to the final wall structure. GP provides data regarding cell wall composition as well as the difficulty in extractability of these components in the wall (thereby revealing the relative tightness of their integration to the wall) [20]. GP has been extensively used in analysing cell walls for functional characterisation of genes involved in cell wall biosynthetic process, pretreated and bio-converted plant biomass, and comparative glycomics of different plant phylogenies based on their cell wall composition [21–23]. However, the limitations with GP are that firstly, low-molecular-weight glycans released by cell wall extractions do not bind to solid supports such as nitrocellulose, glass slides, or multiwell plastic plates. The lower limit of the glycan size while not definitively determined, is greater than 10 kDa. Secondly, certain glycans may get modified by the harsh alkaline extraction conditions, for example, the loss of acetylation or methylation [24].

In situ visualisation by immunolabelling techniques requires the fixation, embedding, and sectioning of biomass samples which are then probed by mAbs followed by a fluorescently tagged secondary antibody allowing an in situ visualisation of glycan epitope distribution under a fluorescence microscope [25]. Immunolabelling has aided in determining epitope distribution of cell wall biosynthetic mutants such as WRKY transcription factor knockouts, xylan-deficient mutants, and compared distribution of glycan epitopes in plant biomass before and after various methods of pretreatments to reduce cell wall recalcitrance [26, 27]. A major disadvantage of in situ visualisation is that any glycan epitopes that are buried within the cell walls (masked) are not exposed by the sectioning process and therefore, are not visualised [25]. In situ immunolocalisation studies along with GP could thus be significantly more powerful as these two methods together provide complementary information [19].

Recent epitope characterisation of xylan-directed mAbs through automated oligosaccharide synthesis provides a library of xylan structural epitopes specifically recognised by monoclonal antibodies (mAbs) [6]. Therefore, with use of different mAbs, it is now possible to monitor abundance throughout most major structural regions of xylans that vary based on their degree of polymerisation (DP), arabinosylation, acetylation, and MeGlcA substitution [6].

Studies with complementary approaches of full-scale GP, immunolabelling, and incorporating the details of above-mentioned recent advances in xylan epitope characterisations provide a powerful method in studying the patterning of xylan deposition in different stages of stem development. Our study, for the first time, describes xylan deposition and its spatio-temporal distribution over different stages of Arabidopsis stem development/maturation through GP, xylan epitope monitoring, and immunolabelling. Moreover, xylan patterns that we identified were supported by in silico gene expression analysis. Our results from GP, xylan epitope monitoring, immunolabelling, and expression data depict that xylan patterning increases throughout stem development. Interestingly, certain xylan epitope structure abundance displays degrees of specificity with regards to certain developmental stages of the stem. Lastly, GP illustrated that xylan epitope abundance and distribution may also influence other patterning and distributions of non-xylan epitopes such as pectins and xyloglucan across stem development. Therefore, using an epitope-directed approach may be useful in further characterising xylan biosynthetic mutants based on their specific xylan epitope phenotypes across developmental gradients of organs.

## Results

### Glycome profiling reveals differences in cell wall glycan epitope distribution and abundancies among sequential extracts throughout Arabidopsis stem development

**Fig. 1:**
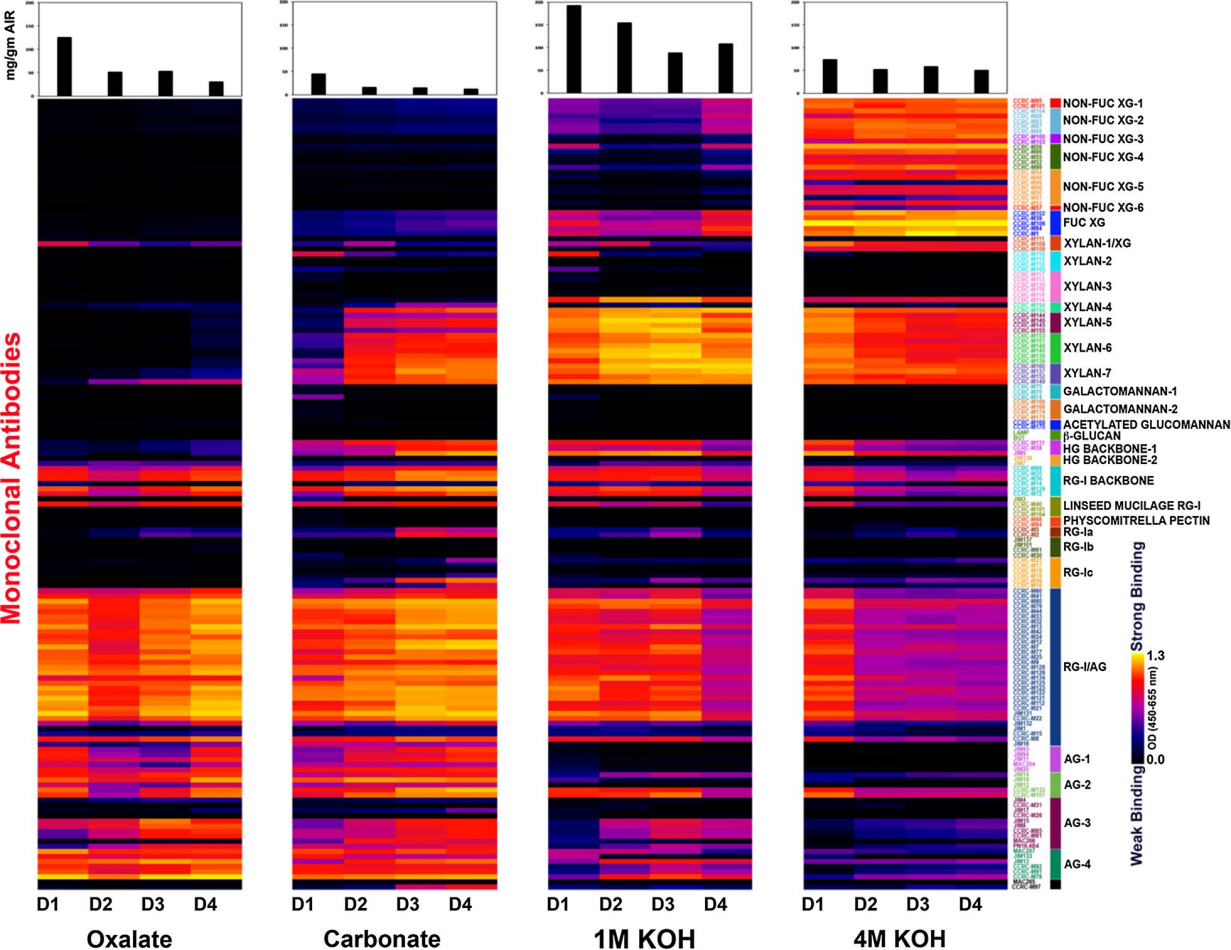
Glycome profiling of cell walls extracted from inflorescence stems at different development stages of *Arabidopsis.* These sequential extracts were screened using 155 mAbs against most major plant cell wall glycans. The ELISA heat map depicts signal binding strength where yellow, red, and black colours represent strong, medium, and no binding, respectively. The groups of mAbs are based on their specificity to different cell wall glycans at the right-hand side of the figure. The top bar graph shows the mg soluble (glucose equivalent) recovered per gram of biomass

*Arabidopsis thaliana* plants grown at identical growth conditions (Additional file 1: Figure S1) were used to perform a developmental gradient dependent study to reveal dynamics in xylan structure and deposition during stem maturation. Arabidopsis inflorescence stems of 24 cm in height were divided into four equal sections and represented by apical (D1), lower apical (D2), upper basal (D3), and basal (D4) sections (Additional file 1: Figure S1) in order to obtain samples representing gradients of stem maturation process. Cell wall materials were isolated from these segments, and sequential extracts from these cell walls were subjected to glycome profiling (see “Methods” section). A comprehensive suite of plant cell wall glycan-directed monoclonal antibodies (mAbs) that could monitor most major non-cellulosic cell wall glycans were employed to perform this analysis (Fig. 1). Most carbohydrate material was recovered from the 1 M KOH fraction, followed by 4 M KOH, carbonate, and oxalate extracts with the exception of apical (D1) developmental stage wherein the second highest amount of material was recovered during oxalate extraction (potentially due to the higher proportion of primary walls during this stage of stem development). Glycome profiling revealed the presence of most major non-cellulosic cell wall glycan epitopes among stem developmental gradients (D1 to D4 segments) mentioned above and how these epitope abundancies varied across various extracts from these gradients (Fig. 1). In the oxalate extracts from D1 through D4 segments, a significant abundance of pectic arabinogalactan and arabinogalactan epitopes, as indicated by the strong binding of mAbs belonging to clades, RG-I/AG and AG-1 through 4, and rhamnogalacturonan-I (RG-I) backbone epitopes, as indicated by the binding of RG-I backbone clade of mAbs, was observed. However, overall patterns of abundancies were subtly different across developmental stages. One notable difference was the reduced abundance arabinogalactan epitopes recognised by AG-1 and AG-2 clades of mAbs in D2 and D3 segments. Again, highest amounts of oxalate-released carbohydrate material was recovered in the D1 segments hinting a significantly higher proportion of primary cell walls in this apical segment causing release of increased proportion of pectic components. In the carbonate extract, baring the trace amounts of non-fucosylated and fucosylated xyloglucans, all other non-cellulosic glycan epitopes detected (including xylan, homogalacturonan, RG-I backbone, pectic arabinogalactan, and arabinogalactan epitopes) exhibited a general trend of increasing abundance as the stem matures (D1 to D4). However, marginally increased amounts of carbohydrates were released from D1 cell walls compared to other segments potentially due to the higher proportion of pectic components originating from the increased presence of primary walls in apical (D1) stems. Following a development-dependent pattern, relative proportion of pectic backbone, pectic arabinogalactan, and arabinogalactan epitopes were significantly reduced in 1 M KOH extract from D4 segment and 4 M KOH extracts from D2, D3, and D4 stages. Xyloglucan epitopes were detected in 1 M and 4 M KOH extracts from all stem developmental regions. In 1 M KOH extracts, a marginally reduced proportion of xyloglucans were observed in D2 and D3 segments. However, significantly higher abundance of xyloglucan epitopes was obviously detected in 4 M KOH across all extracts from all segments. Since the focus of this study is delineating xylan composition, structure, extractability, and deposition on the wall as a function of stem development, we conducted specifically focussed analyses employing the subset (that were generated employing the whole spectrum of fully characterised xylan-directed mAbs) of this whole glycome dataset pertaining to xylans (Fig. 1), the results of which are described in the subsequent sections.

### Xylan-focussed epitope profiling reveals varied patterns in deposition of xylan sub-structures across stem development in Arabidopsis

We specifically focussed on the patterns of xylan epitope abundance and extractabilities among the four cell wall extracts isolated from different developmental regions of stem, D1–D4. We wanted to take advantage of the monospecific and well-defined epitope information that is currently available for all xylan-directed antibodies belonging to the groups Xylan-4 through Xylan-7, allowing advanced molecular level monitoring of xylan structures [6].

**Fig. 2:**
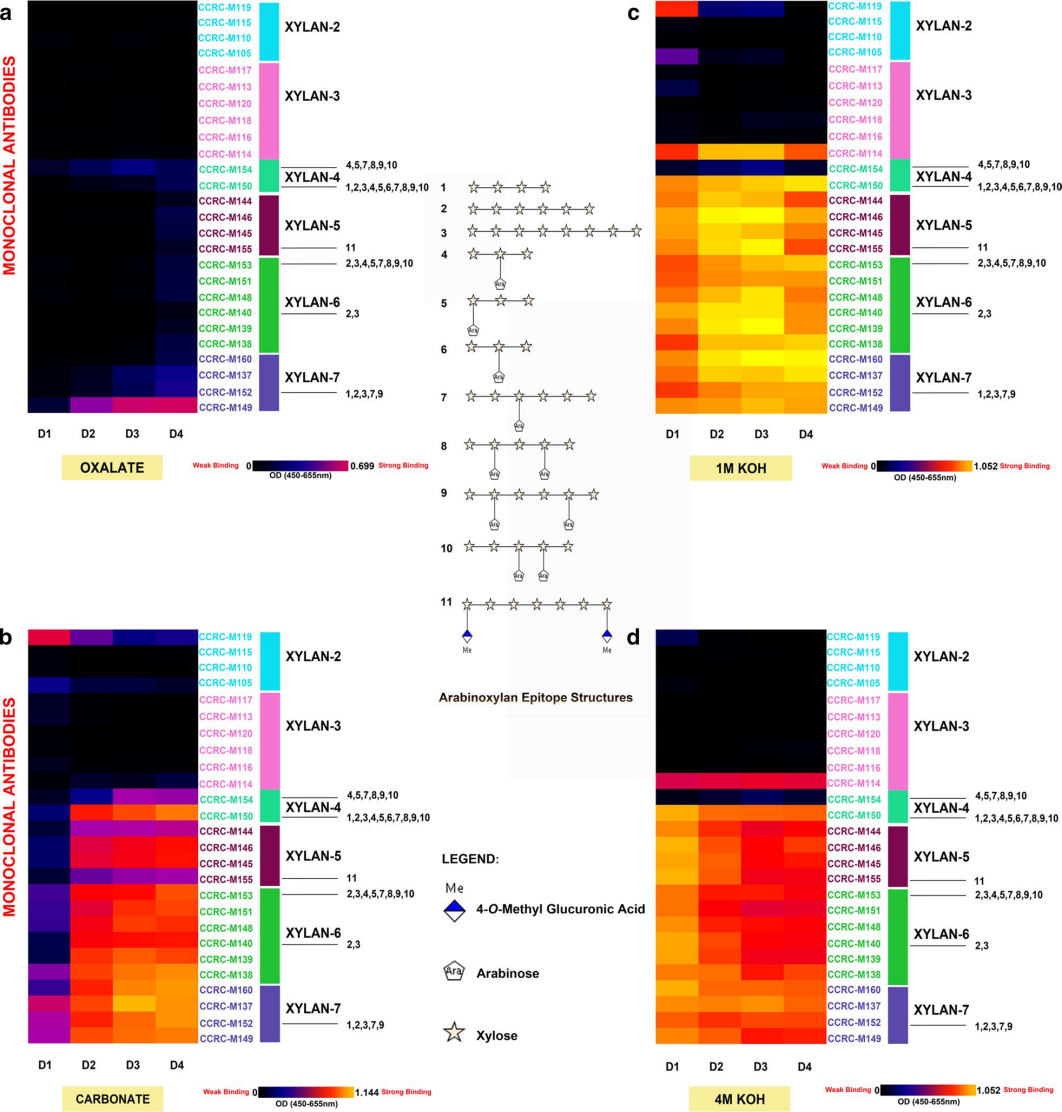
Xylan profiling of Col-0 inflorescence stems. ELISA binding signals specific to xylan epitope groups (Xylan2 to Xylan7) were isolated from this figure to depict distinct xylan epitopes enriched from different chemical extracts (**a** oxalate; **b** carbonate; **c** 1M KOH; **d** 4M KOH) with increasing harshness and at different stages (D1-D4) of *Arabidopsis* stem development. The ELISA heat map depicts signal binding strength where yellow, red, and black colours represent strong, medium, and no binding, respectively. The groups of mAbs are based on their specificity to different xylans at the right hand side of the figure. The top bar graph displays the mg soluble (glucose equivalent) recovered per gram of biomass. The middle illustration depicts the specific xylan epitope structures that xylan-directed specific mAbs bind to. Xylan epitope characterisation was based on the results of Schmidt et al. [6]

Figure 2 depicts the results of xylan epitope profiling of these developmental regions of Arabidopsis stem. Previous studies from our laboratory had broadly grouped xylan-directed mAbs into 7 clades (xylan-1 through xylan-7 clades) based on the hierarchical clustering of ELISA binding response data of these mAbs against 55 structurally defined plant polysaccharides [28]. More recent studies, making a significant scientific advance in the field, identified 11 structural regions of xylan (epitopes) as shown in the figure that are monospecifically recognised by antibodies belonging to clades Xylan-4 through Xylan-7 [6]. In a broad sense, as depicted in Fig. 2, we currently have well-defined antibodies that could detect small degree of polymerisation (DP) homoxylan regions (DP, 3–5) (CCRC-M150, CCRC-M152, CCRC-M153, and CCRC-M154), larger DP homoxylan regions (DP, 4–8) (CCRC-M140, CCRC-M150, and CCRC-M152), single arabinosyl-substituted xylan backbone regions (CCRC-M150, CCRC-M152, CCRC-M153, and CCRC-M154), double arabinosyl-substituted xylan backbone regions (CCRC-M150, CCRC-M152, CCRC-M153, and CCRC-M154), and MeGlcA-substituted xylan regions (CCRC-M155). Taken together, analyses using mAbs against this broad repertoire of xylan epitopes could allow monitoring of most major xylan structures among higher plants.

In the oxalate extracts from all segments, the only xylan epitope significantly detected was the CCRC-M149-recognised epitope (small DP homoxylan and High DP xylan). This epitope showed an increase in its abundance in oxalate extract as the stem matures, thus maximum abundance is attained at the D4 segment stage. In carbonate extracts, however, xylan epitopes recognised by Xylan-4 to Xylan-7 clades of antibodies were significantly abundant in D3–D4 segments (except epitopes recognised by CCRC-M154 of Xylan-4 clade; CCRC-M144 and CCRC-M155 of xylan 5 clade that showed only marginal abundance). On the other hand, only Xylan-7 epitopes were present in detectable levels at the apical (D1) stage. Furthermore, the abundance of all xylan epitopes showed a general increasing trend as stem matured. Interestingly, only in the apical stage of stem development, xylan epitopes recognised by CCRC-M119 were significantly present. In 1 M and 4 M KOH extracts, xylan epitopes recognised by CCRC-M114 of Xylan-3 clade, CCRC-M150 of Xylan-4 clade, and all antibodies belonging to Xylan-5 through Xylan-7 groups were abundantly present. However, their patterns of extractability varied depending on the developmental regions. For instance, comparatively, the highest abundance of these epitopes is in 1 M KOH extracts and was observed in D2 and D3 stages. In 4 M KOH extracts, the highest noted epitope abundance occurred at the D1 stage. This could be due to the increased formation of secondary walls through lignification in mature stems which could result in lower extractability of directly or indirectly lignin-associated cell wall components including xylan.

We observed that xylan deposition exhibits a patterning that is dependent on the developmental stages of the inflorescence stem in Arabidopsis. Based on oxalate, carbonate, and 1 M KOH extracts, we observed an increase in xylan epitope abundance in these extracts as stem matures, hinting the enhanced biosynthesis and deposition of xylan. In the oxalate extractible material, unsubstituted xylan epitopes showed an increase as stem matures as shown by the increase in signal of CCRC-M149 (Fig. 2). 4 M KOH extracts displayed decreasing xylan epitope proportions as stem development progressed from D1 to D4 stages. D1 stage showed the highest 4 M KOH extractible xylan epitope abundance which can be possibly attributed to a significant degree of association between xylan and pectin in the predominantly present primary walls. While xylan epitopes were shown to be decreasing in proportion as the stem develop in 4 M KOH extracts, concomitant increase in xyloglucan epitope abundance was observed. Therefore, the results point to a net increase in hemicellulose epitopes as stem development occurs in Arabidopsis.

### Xylan immunolabelling of stems across different developmental stages

**Fig. 3:**
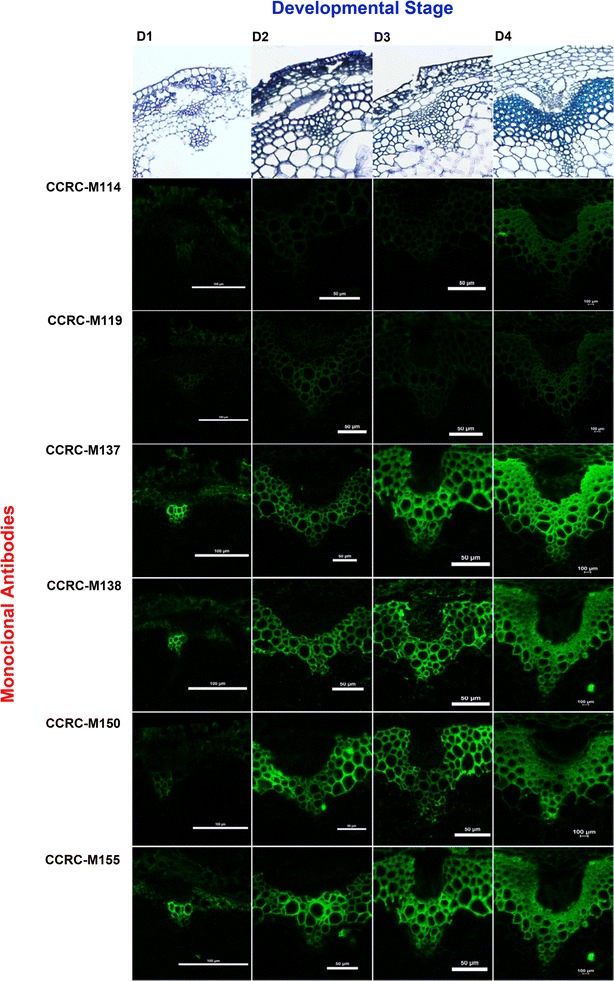
Immunolabelling of xylan epitopes of Col-0 stems at different development stages (D1-D4). Top row: toluidine blue-O staining of stems to show anatomical differences of vasculature tissue at different developmental stages. Equivalent sections were immunolabelled with xylan-directed mAbs CCRC-M114, CCRC-M119, CCRC-M137, CCRC-M138, CCRC-M150, and CCRC-M155. CCRC-M114 and CCRC-M119 show little to no signal and CCRC-M137 to CCRC-M155 show increasing signal throughout stem development

*Arabidopsis* inflorescence stems were sectioned, paraffin-embedded, deparaffinised, treated with 0.1 M KOH for 15 min, and immunolabelled according to methods by Avci et al. [25]. 0.1 M KOH treatment was performed because untreated sections produced little or no signal in all sections of all the developmental stages sampled. Treatment with 0.1 M KOH sufficiently removed modifications on the glycan epitopes present in the section to allow for adequate binding with the selected xylan mAbs. We selected these specific antibodies from the different xylan groups represented (Xylan-2 to Xylan-7) based on their abundance levels from the xylan-specific glycome profile (Fig. 2). Our immunolabelling results showed that xylan epitopes display increasing abundance throughout stem development in Arabidopsis. However, certain xylan epitopes, particularly those recognised by CCRC-M119 and CCRC-M114 are only present at later stages of stem development, towards the basal portion of the stem (D4) (Fig. 3). In situ visualisation of Arabidopsis stems using immunolabelling using the group of selected mAbs that bind to specific xylan epitope confirmed the results of our glycome profile revealing that hemicellulosic epitopes increases as stem development progresses.

### In silico analysis of putative and proven xylan biosynthesis genes and enzymes has similar patterns of expression during development to glycome profiling using xylan-directed epitopes

To determine whether our observed glycome profiling and immunolabelling patterns were due to developmentally regulated differential expression of xylan biosynthetic genes or from developmentally regulated post-translational modification of enzyme products, we performed in silico expression analysis using a publicly available microarray database of the known putative and proven xylan biosynthesis genes and enzymes to determine whether or not their expression pattern shares similarities with our xylan epitope profile. We accessed the Expression Browser database from the Botany Array Resource and selected the AtGenExpress developmental dataset [29]. From this, we selected the relative gene expression output option that used the average gene expression of replicate treatments relative to the appropriate control. We then surveyed the relative gene expression data of known xylan genes that were available in this dataset and created an output list of 15 xylan genes (Additional file 2: Figure S2). The gene expression data displayed overall patterns similar to the xylan-specific glycome profile and immunolabelling data in that most genes increased throughout developmental stages with their relative expression highly expressed at later stages of stem development. However, we also observed that certain selected xylan genes do not display increasing patterns of expression towards later stages of stem development. For example, IRX9-L, GUX4/5, and GXM2, showed variable expression changes in all surveyed stages of stem developments. The expression patterns of these particular genes may not fully represent the patterns obtained from our immunolabelling results because the list of chosen mAbs only represented a handful of xylan epitopes. Therefore, any of the mAbs that recognised xylan epitopes corresponding to the expression patterns of IRX9-L, GUX4/5, and GXM2 may not have been represented. IRX9-L, a functional homologue of IRX9 belonging to the glycosyltransferase (GT) 43 family is known to play a minor redundant role in GX backbone biosynthesis when compared to IRX9 [30]. A study by Wu et al. [30] demonstrated that *irx9*-*L* alleles displayed growth and secondary cell wall formation phenotypes similar to wild-type. Additionally, IRX9-L was indicated to perform non-essential redundant functions with IRX9. Hence, this may explain why the variable gene expression changes observed in our in silico analysis were not fully represented with the corresponding immunolabelling data. GUX4 and GUX5 are GT 8 family proteins that may have putative roles in glucuronosyl substitutions onto the xylan backbone. Studies using GlcA transferase (GlcAT) assays demonstrated that GUX5 but not GUX4 had GlcAT activities [17, 31]. However, mAb epitope characterisation specific to xylans with GlcA substitutions has not yet been established. Therefore, any GlcA substitutions added to the xylan backbone as a result of differential expression of GUX4 and GUX5 genes during different stages of stem development would not be detected using immunolabelling. Lastly, GXM2 is a DUF579 domain-containing protein known to be implicated in glucuronoxylan methylation. Recent genetic and chemical analysis has revealed that GXM2, along with GXM1 and GXM3 are methyltransferases involved in 4-*O*-methylation of GlcA sidechains. Furthermore, the analyses demonstrated that GXM1, GXM2, and GXM3 are functionally redundant glucuronoxylan methyltransferases and among them, GXM3 is the predominant gene responsible for GlcA methylation [32]. This may explain why the immunolabelling signal intensity of CCRC-M155, which specifically detects xylan epitopes containing 4-*O*-MeGlcA substitutions, corresponds to the expression pattern of GXM3. It should also be noted however, that we cannot confirm whether or not the expression data of the xylan genes surveyed that correspond to our glycome profile and immunolabelling results are directly responsible for the observed distribution of xylan epitopes. In order to do so, we are currently conducting developmentally induced variation in xylan biosynthetic genes at the transcript levels using RNA-seq methods. Once these studies are completed we intend to combine gene transcript studies with their corresponding available glycome datasets.

## Discussion

Plants cell walls are structurally highly complex, heterogeneous, and vary significantly across species, organs, and development. The studies herein analysed variations in cell wall makeup during Arabidopsis stem maturation process, especially focussing on xylan integration into the walls. Previous studies have demonstrated organ development-dependent variations in cell wall makeup in diverse phylogenetic classes of plants such as willow (*S. purpurea*) and switchgrass [12, 18]. However, more comprehensive and rapid approaches employing advanced and reliable cell wall analytical tools that can operate in a medium-to-high-throughput manner have always been in demand. In the current study, we developed, validated, and report such an advanced immunological approach for higher plant xylan analyses employing a comprehensive collection of fully characterised xylan-directed mAbs. Agreeing with the above-mentioned studies on willow and switchgrass, the whole glycome profiles (Fig. 1) of cell walls isolated from different development stages of stems revealed changes in the composition and extractabilities of most major non-cellulosic cell wall matrix glycans revealing unique cell wall makeup for different developmental stages of an organ. Of the comprehensive suite of cell wall directed mAbs used in the glycome profiling analysis, monospecific epitope-level structural information is currently available for most major xylan-directed antibodies [6]. Together, these xylan-directed antibodies could monitor five groups of xylan epitope structural regions namely small DP (DP, 3–5) homoxylan, large DP (DP, 4–8) homoxylan, single arabinosyl-substituted regions, double arabinosyl-substituted regions, and 4-*O*-MeGlcA-substituted regions. This allows monitoring of most major structural regions of complex xylans in higher plants including unsubstituted, arabinosyl-substituted, and glucurono/methylglucurono-substituted xylan structures. Given this extent of monospecific epitope-level information available for such a large group of xylan-directed antibodies, a unique opportunity opens up towards a molecular level understanding of xylan formation and integration into cell walls under diverse spatio-temporal stages of plant organs. In this work, we wanted to take advantage of the above well-characterised xylan-directed probes to study Arabidopsis stem development, validate the same, and develop an advanced tool for rapid and reliable characterisation of xylan formation and integration to the wall in higher plants.

Our results showed that in Arabidopsis, xylan incorporation is initiated early on in the vascular tissue development; however, the integration of the different xylan epitopes surveyed varies across developmental stages of the stem. This is clearly demonstrated by the altered extractability of specific xylan epitopes from cell walls isolated in different developmental regions. Summarising the glycome profile results of cell walls from D1 (youngest, apical) to D4 (most mature, basal) segments, we generated a table depicting the detection of the five above-mentioned epitope classes of xylan among extracts (Table 1). Oxalate extracts contained only homoxylan epitopes in mature (D3-D4) segments but arabinosyl-substituted xylan epitopes are present in traces (hence we put a minus sign in the table) starting at the intermediate stem segments (D2). In carbonate extracts, both substituted and unsubstituted xylan epitopes are negligibly present in the youngest tissue (D1), indicating that loosely integrated xylans are present but to a lesser extent in young developmental stages. These results indicate that there exist sub-classes of xylan which are loosely integrated to the cell walls and hence being extracted by least harsh reagents such as oxalate and carbonate. These loosely integrated classes of xylan do exhibit variations across developmental stages. Furthermore, these classes of xylan seem to contain unsubstituted and substituted structural regions of xylan. These loosely integrated classes of xylan, however, in the apical (D1) region seems to predominantly contain unsubstituted xylan regions with significantly lesser amounts of substituted xylan regions. Our study shows that the highest degree of variation in xylan structure and integration are exhibited by loosely bound xylan components across developmental stages of stem.

**Table 1:** Xylan epitope patterning observed in different extracts across developmental stages of Arabidopsis based on Fig. 3

Xylan epitope	Oxalate				Carbonate				1 M KOH				4 M KOH			
	D1	D2	D3	D4	D1	D2	D3	D4	D1	D2	D3	D4	D1	D2	D3	D4
Small DP (3–5)	−	−	−	+	+	+	++	++	+	+	+	+	+	+	+	+
Large DP (4–8)	−	−	+	+	+	++	++	++	++	++	+++	+++	+++	++	++	++
Single Arabinosyl residue	−	−	−	−	+	+	++	++	++	++	+++	+++	+++	++	++	++
Double Arabinosyl residue	−	−	−	−	+	++	++	++	++	++	+++	+++	+++	++	++	++
4-*O*-methyl glucuronic acid	−	−	−	−	−	+	+	+	++	++	+++	++	+++	++	++	++

DP, degree of polymerisation; −, +, ++, and +++ refers to: absent, present, abundant, and highly abundant, respectively

Glycome profiling results demonstrated that all five xylan epitope categories are significantly present in 1 M and 4 M KOH extracts of cell walls from all developmental regions. These high alkali-extracted xylan components collectively represent tightly integrated xylan classes. These tightly integrated classes of xylan also contain unsubstituted and substituted regions (indicated by the binding of all xylan-4 through 7 clades of mAbs). All epitopes of tightly integrated xylan are abundantly present in all developmental stages including the youngest (D1) stage revealing lesser degree of variation of these classes of xylan across development. However, for tightly integrated xylan classes, subtle variations in patterns were observed in 1 M KOH extracts in that an increasing abundance of both unsubstituted and substituted regions of xylan was noted with the increasing stem maturation. In contrast, 4 M KOH extracts displayed decreasing abundance of these xylan epitopes as stem development progressed. This can be attributed to the corresponding increase in the proportion of xyloglucan epitopes in 4 M KOH extracts as shown in Fig. 1.

Immunolabelling analysis conducted in our study largely agreed with the glycome profiling results in that an increased intensity of labelling was observed during stem maturation. However, our results contradict those previously gathered by Kim and Daniel using xylan-directed LM10 and LM11 mAbs. They obtained different xylan epitope distributions compared to our study [33]. Their study selected LM10, which displays specificity towards unsubstituted or low-substituted xylans, and LM11, a wheat arabinoxylan and unsubstituted xylan-specific antibody [34]. Using these antibodies, their results displayed no differences in immunolabelling intensities in vessels and fibres at the apical and basal stem portions of Arabidopsis. Our results on the other hand, clearly demonstrated that certain homo-xylan epitopes (recognised by CCRC-M137, CCRC-M138, and CCRC-M150) displayed increasing intensities as stem development progressed. Additionally, epitopes that bind to CCRC-M114 and CCRC-M119 mAbs are absent in early (D1) and intermediate (D2) stages, but appear only in mature stem segments (D4). There are critical differences between these two studies, in our study, we surveyed a more comprehensive representation of stem developmental gradients including four regions namely apical (D1), lower apical (D2), upper basal (D3), and basal (D4). Again, in our current study, we used a more comprehensive repertoire of mAbs whose epitopes are well-defined. Hence, a direct comparison between these two studies may not be feasible. Lastly, our whole glycome profiling, xylan epitope-directed profiling, and in silico expression analysis of xylan genes [29] are also contradictory to the transcriptomic studies performed by Minic et al. [35], suggesting that xylan deposition starts to occur at the intermediate and late stages of stem development.

We represented the gravimetric amounts of material extracted out during each extraction step and the amounts of carbohydrate materials decreased with increasing stem maturity. This is because, as the stem matures, the secondary cell wall formation increases with increasing lignification processes. Hence, the extractability of non-cellulosic matrix glycans is reduced due to the increased abundance of more structurally rigid secondary walls. One of the main objectives of this study is to optimise a tool for dissecting xylan epitope extractability from different developmental zones of stem and use this tool to conduct comparative glycomics analysis of comparable developmental regions of organs originating from cell wall biosynthetic mutants in Arabidopsis and/or conduct such studies across plants belonging to diverse phylogenetic classes. Studies using this tool would thus allow us to compare and correlate abundancies of diverse xylan epitope structures among cell wall extracts prepared from comparable organ developmental zones. The two approaches employed here provide complementary information on the cell wall epitope abundance monitoring in vitro and in vivo changes. The two approaches (glycome profiling and immunolabelling) measure glycan epitopes differently. Glycome profiling monitors the chemically extracted glycan epitopes which are mostly not masked by other cell wall components. However, in immunolabelling, in vivo distribution of the epitopes is monitored which still could be conformationally masked (even after 0.1 M KOH treatment) hence a direct correlation between glycome profiling and immunolabelling data is not expected. Additionally, subjecting the cell wall to chemical fractionation may cause modification/s of some glycans resulting in the loss of some epitope structures [19, 20]. For example, in glycome profiling, CCRC-M114 displays increasing epitope abundance in all stages. However, the immunolabelling data for this specific epitope show signals only at the basal stage of the stem. Additionally, the similar increasing pattern of epitope abundance specific to CCRC-M147 in glycome profiling and immunolabelling is not significantly correlated. Thus, complementary information provided by these two approaches become invaluable when conducting comparative glycomic studies [19, 20].

Our systematic approach allows the comprehensive monitoring of the spatial distribution of specific xylan structures that occurs during stem development. By combining glycome profiling, immunolabelling techniques, and the available xylan epitope characterisation data, we can apply such an approach in order to explain certain behaviours in wood formation, an important potential source of raw materials for biofuel production [2, 36]. Applying our approach to surveying the structural and compositional differences observed in xylan biosynthetic mutants can reveal new insights into the functional characterisation of the complete spectrum of genes required for xylan biosynthesis/modification in the context of stem development [37]. Furthermore, our approach is a rapid, cost effective, and is a high-throughput method to determine the effects of pretreatments on xylan and other hemicellulosic structures in biofuel crops without requiring time-consuming and sophisticated computational methods involved in NMR and FTIR approaches [38–40].

## Conclusion

Our method of xylan epitope-directed glycome profiling complemented by in situ visualisation using immunolabelling provides a viable approach to characterise specific xylan epitopes that are deposited in the cell wall at different stages of stem development. Furthermore, our approach can monitor the changes in xylan epitope composition throughout stem development that are caused by specific mutations among xylan biosynthetic/modifying genes. The approach could also be a powerful tool in an evolutionary context in that phylogenetic analyses of variations in xylan structures across species, organ, and developmental stages can be performed in a rapid and medium--to-high-throughput manner. This approach also opens up the possibility to employ other cell wall glycan-directed mAbs for monitoring other important cell wall glycans such as pectins, arabinogalactans, and xyloglucans.

## Methods

### Growth and harvesting

Thirty-two wild-type *Arabidopsis thaliana* ecotype Columbia plants were germinated on 0.8% w/v agar plates containing MS nutrients and B5 vitamins for 2 weeks prior to being transferred to compost containing vermiculite and perlite (10:1:1). Plants were subsequently grown at 22 °C in controlled-environment cabinets using short-day (8-h light/16-h dark) followed by long-day (16-h light/8-h dark) conditions. Arabidopsis inflorescence stems were grown to a height of 22–25 cm and were approximately divided into four equal segments from top to bottom to represent the different stages of stem development.

### Biological material

24-cm inflorescence stems cut into 6-cm equal segments and were flash-frozen using liquid nitrogen. The segments were subsequently ground with a mortar and pestle. 200 mg ground tissue was then transferred to a 50 ml tube for alcohol insoluble residues (AIR) that were prepared as previously described [20]. Glycome profiling of these AIR preparations was performed using the method described in Pattathil et al. [20]. In brief, glycome profiling involved preparing cell wall extracts using increasingly harsh reagents (Ammonium oxalate, sodium carbonate, 1 M KOH, and 4 M KOH) and subsequent enzyme-linked immunosorbent assay (ELISA) screening of these extracts using a comprehensive suite of plant cell wall glycan-directed monoclonal antibodies (mAbs). Plant glycan-directed mAbs were from laboratory stocks (CCRC, JIM, and MAC series) at the Complex Carbohydrate Research Center (available through CarboSource Services; http://www.carbosource.net) or were obtained from BioSupplies (Australia) (BG1, LAMP). Additional information about the mAbs employed in glycome profiling can be seen in Additional file 3: Table S1.

### Histology

Inflorescence stem segments were fixed for 1 h in ice-cold 100% acetone and rotated (20 rpm). The acetone was removed and replaced with fresh ice-cold 100% acetone and rotated (20 rpm) overnight at 4 °C. Samples were then passed through 3:1, 1:1, and 1:3 gradients of acetone:histo-clear for 1 h each followed by an extra 100% histo-clear change. Tissue samples were incubated at 57 °C for Paraplast (wax) infiltration. Tissue blocks with appropriate size and orientation were sectioned using a microtome in order to obtain 5-μm-thick sections; sections were subsequently placed on charged glass slides and dried at 45 °C for 30 min. For anatomical observations, sections were stained with 0.025% toluidine blue (2 min). After staining, sections were dehydrated with 75% (v/v) ethanol. Sections were observed under a compound microscope with bright-field illumination.

### Immunolabelling

Stem sections were treated with 0.1 M KOH with 10 mM NaBH_4_ for 15 min and were rinsed with ddH2O three times. Immunolabelling was performed as described previously [25]. Labelling was visualised using an Eclipse 80i light microscope (Nikon, Melville, NY) equipped with epifluorescence optics and Nikon B-2E/C filter. Images were captured using a Nikon DS-Ri1 camera head (Nikon, Melville, NY) and NIS-Elements Basic Research software. Images were assembled without further processing using Adobe Photoshop (Adobe Systems, San Jose, CA).

## Additional files

**Additional file 1: Figure S1.** *Arabidopsis thaliana* Col-0 background stems grown to ~24 cm for stem harvesting.

**Additional file 2: Figure S2.** Heat map showing gene expression from publicly available microarray data [29] of xylan genes in different stages of Col-0 stem development from immature (D1), intermediate (D2-D3), and mature (D4) stems. Levels of low (white) and high expression (red) are shown on a log2 scale for each xylan gene.

**Additional file 3: Table S1.** Detailed list of cell wall glycan-directed monoclonal antibodies (mAbs) used in glycome profiling analyses. The groupings of antibodies are based on the hierarchical clustering of ELISA data generated from screening all mAbs against a comprehensive panel of plant polysaccharide preparations [20, 24] which clusters mAbs according to their predominant polysaccharides recognition patterns. The majority of the listings link to the Wall*Mab*DB plant cell wall monoclonal antibody database (http://www.wallmabdb.net) which provides the detailed descriptions for each mAb, including immunogen, antibody isotype, epitope structure (to the extent known), supplier information, and related literature citations.

## Abbreviations

GAX: glucuronoarabinoxylan
Xyl: xylose
XyG: glucuronoxylan
GlcA: glucuronic acid
mAbs: monoclonal antibodies
GP: glycome profiling
DP: degree of polymerisation
MeGlcA: methyl glucuronic acid
RG-I: rhamnogalacturonan-I
