# Supplementary material for: Xylan epitope profiling: an enhanced approach to study organ development-dependent changes in xylan structure, biosynthesis, and deposition in plant cell walls
Source: Biotechnol Biofuels. 2017 Nov 30;10:245. doi: 10.1186/s13068-017-0935-5 (PMC5707906; doi:10.1186/s13068-017-0935-5)
Supplement: Supplementary file 1 — Additional file 1: Figure S1. Arabidopsis thaliana Col-0 background stems grown to ~24 cm for stem harvesting. [file 13068_2017_935_MOESM1_ESM.docx]

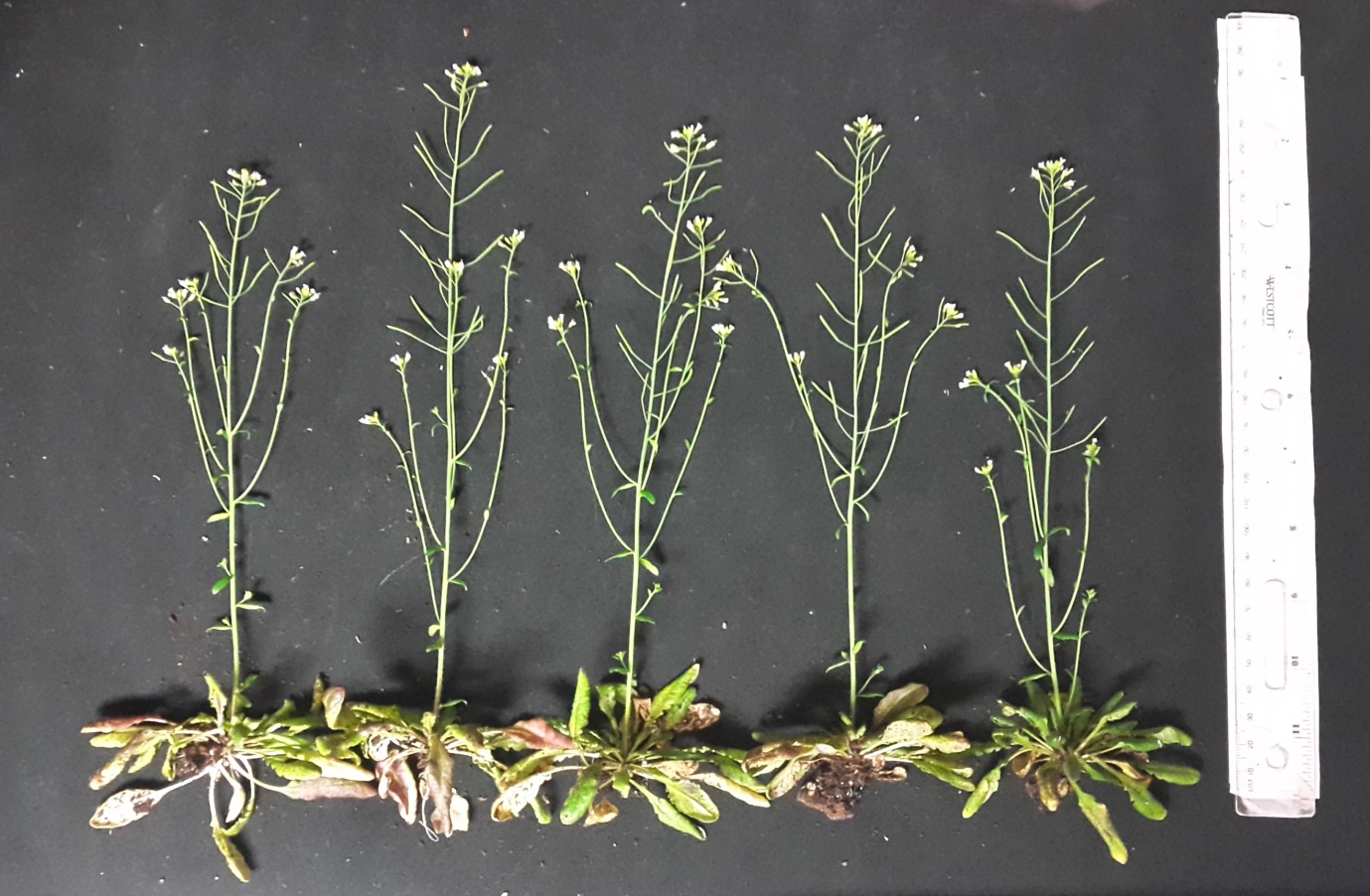


Supplementary Figure 1: *Arabidopsis thaliana* Col-0 background stems grown to ~24cm for stem harvesting.
