# Supplementary material for: Xylan epitope profiling: an enhanced approach to study organ development-dependent changes in xylan structure, biosynthesis, and deposition in plant cell walls
Source: Biotechnol Biofuels. 2017 Nov 30;10:245. doi: 10.1186/s13068-017-0935-5 (PMC5707906; doi:10.1186/s13068-017-0935-5)
Supplement: Supplementary file 2 — Additional file 2: Figure S2. Heat map showing gene expression from publicly available microarray data [29] of xylan genes in different stages of Col-0 stem development from immature (D1), intermediate (D2-D3), and mature (D4) stems. Levels of low (white) and high expression (red) are shown on a log2 scale for each xylan gene. [file 13068_2017_935_MOESM2_ESM.docx]

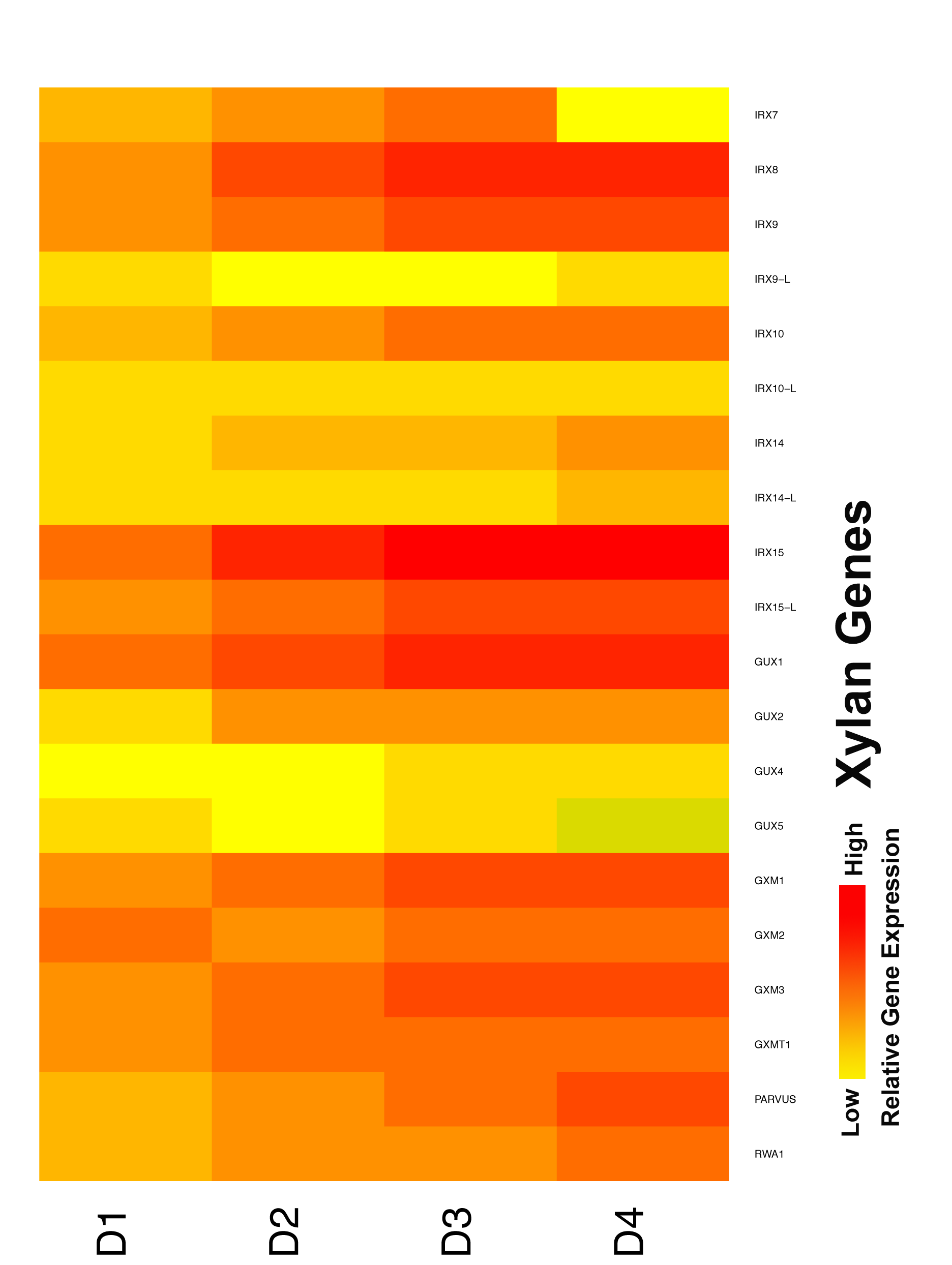


Supplementary Figure 2: Heat map showing gene expression from publicly available microarray data (Toufighi et al., 2005) of xylan genes in different stages of Col-0 stem development from immature (D1), intermediate (D2-D3), and mature (D4) stems. Levels of low (white) and high expression (red) are shown on a log2 scale for each xylan gene.
