# Supplementary material for: Xylan epitope profiling: an enhanced approach to study organ development-dependent changes in xylan structure, biosynthesis, and deposition in plant cell walls
Source: Biotechnol Biofuels. 2017 Nov 30;10:245. doi: 10.1186/s13068-017-0935-5 (PMC5707906; doi:10.1186/s13068-017-0935-5)
Supplement: Supplementary file 3 — Additional file 3: Table S1. Detailed list of cell wall glycan-directed monoclonal antibodies (mAbs) used in glycome profiling analyses. The groupings of antibodies are based on the hierarchical clustering of ELISA data generated from screening all mAbs against a comprehensive panel of plant polysaccharide preparations [20, 24] which clusters mAbs according to their predominant polysaccharides recognition patterns. The majority of the listings link to the WallMabDB plant cell wall monoclonal antibody database (http://www.wallmabdb.net) which provides the detailed descriptions for each mAb, including immunogen, antibody isotype, epitope structure (to the extent known), supplier information, and related literature citations. [file 13068_2017_935_MOESM3_ESM.docx]

**Supplementary Table 1:** Detailed list of cell wall glycan-directed monoclonal antibodies (mAbs) used in glycome profiling analyses. The groupings of antibodies are based on the hierarchical clustering of ELISA data generated from screening all mAbs against a comprehensive panel of plant polysaccharide preparations (Pattathil et al., 2010; Pattathil et al., 2012) which clusters mAbs according to their predominant polysaccharides recognition patterns. The majority of the listings link to the Wall*Mab*DB plant cell wall monoclonal antibody database ([http://www.wallmabdb.net](http://www.wallmabdb.net/)) which provides the detailed descriptions for each mAb, including immunogen, antibody isotype, epitope structure (to the extent known), supplier information, and related literature citations.

**Glycan Group Recognized mAb Names**

| Non-Fucosylated Xyloglucan-1 | [CCRC-M95](http://glycomics.ccrc.uga.edu/wall2/jsp/abdetails.jsp?abnumber=162&amp;abname=CCRC-M95) |
| --- | --- |
|  | [CCRC-M101](http://glycomics.ccrc.uga.edu/wall2/jsp/abdetails.jsp?abnumber=163&amp;abname=CCRC-M101) |
|  |  |
|  | [CCRC-M104](http://glycomics.ccrc.uga.edu/wall2/jsp/abdetails.jsp?abnumber=164&amp;abname=CCRC-M104) |
|  | [CCRC-M89](http://glycomics.ccrc.uga.edu/wall2/jsp/abdetails.jsp?abnumber=160&amp;abname=CCRC-M89) |
| Non-Fucosylated  Xyloglucan-2 | [CCRC-M93](http://glycomics.ccrc.uga.edu/wall2/jsp/abdetails.jsp?abnumber=161&amp;abname=CCRC-M93) |
|  | [CCRC-M87](http://glycomics.ccrc.uga.edu/wall2/jsp/abdetails.jsp?abnumber=158&amp;abname=CCRC-M87) |
|  | [CCRC-M88](http://glycomics.ccrc.uga.edu/wall2/jsp/abdetails.jsp?abnumber=159&amp;abname=CCRC-M88) |
|  |  |
| Non-Fucosylated Xyloglucan-3 | [CCRC-M100](http://glycomics.ccrc.uga.edu/wall2/jsp/abdetails.jsp?abnumber=114&amp;abname=CCRC-M100) |
|  | [CCRC-M103](http://glycomics.ccrc.uga.edu/wall2/jsp/abdetails.jsp?abnumber=113&amp;abname=CCRC-M103) |
|  |  |
|  | [CCRC-M58](http://glycomics.ccrc.uga.edu/wall2/jsp/abdetails.jsp?abnumber=155&amp;abname=CCRC-M58) |
|  | [CCRC-M86](http://glycomics.ccrc.uga.edu/wall2/jsp/abdetails.jsp?abnumber=157&amp;abname=CCRC-M86) |
| Non-Fucosylated  Xyloglucan-4 | [CCRC-M55](http://glycomics.ccrc.uga.edu/wall2/jsp/abdetails.jsp?abnumber=148&amp;abname=CCRC-M55) |
|  | [CCRC-M52](http://glycomics.ccrc.uga.edu/wall2/jsp/abdetails.jsp?abnumber=145&amp;abname=CCRC-M52) |
|  | [CCRC-M99](http://glycomics.ccrc.uga.edu/wall2/jsp/abdetails.jsp?abnumber=152&amp;abname=CCRC-M99) |

|  | [CCRC-M54](http://glycomics.ccrc.uga.edu/wall2/jsp/abdetails.jsp?abnumber=147&amp;abname=CCRC-M54) |
| --- | --- |
|  | [CCRC-M48](http://glycomics.ccrc.uga.edu/wall2/jsp/abdetails.jsp?abnumber=77&amp;abname=CCRC-M48) |
|  | [CCRC-M49](http://glycomics.ccrc.uga.edu/wall2/jsp/abdetails.jsp?abnumber=76&amp;abname=CCRC-M49) |
| Non-Fucosylated  Xyloglucan-5 | [CCRC-M96](http://glycomics.ccrc.uga.edu/wall2/jsp/abdetails.jsp?abnumber=151&amp;abname=CCRC-M96) |
|  | [CCRC-M50](http://glycomics.ccrc.uga.edu/wall2/jsp/abdetails.jsp?abnumber=143&amp;abname=CCRC-M50) |
|  | [CCRC-M51](http://glycomics.ccrc.uga.edu/wall2/jsp/abdetails.jsp?abnumber=144&amp;abname=CCRC-M51) |
|  | [CCRC-M53](http://glycomics.ccrc.uga.edu/wall2/jsp/abdetails.jsp?abnumber=146&amp;abname=CCRC-M53) |
|  |  |
| Non-Fucosylated Xyloglucan-6 | [CCRC-M57](http://glycomics.ccrc.uga.edu/wall2/jsp/abdetails.jsp?abnumber=154&amp;abname=CCRC-M57) |
|  |  |
|  | [CCRC-M102](http://glycomics.ccrc.uga.edu/wall2/jsp/abdetails.jsp?abnumber=142&amp;abname=CCRC-M102) |
|  | [CCRC-M39](http://glycomics.ccrc.uga.edu/wall2/jsp/abdetails.jsp?abnumber=78&amp;abname=CCRC-M39) |
| Fucosylated  Xyloglucan | [CCRC-M106](http://glycomics.ccrc.uga.edu/wall2/jsp/abdetails.jsp?abnumber=112&amp;abname=CCRC-M106) |
|  | [CCRC-M84](http://glycomics.ccrc.uga.edu/wall2/jsp/abdetails.jsp?abnumber=124&amp;abname=CCRC-M84) |
|  | [CCRC-M1](http://glycomics.ccrc.uga.edu/wall2/jsp/abdetails.jsp?abnumber=1&amp;abname=CCRC-M1) |
|  |  |
|  | [CCRC-M111](http://glycomics.ccrc.uga.edu/wall2/jsp/abdetails.jsp?abnumber=168&amp;abname=CCRC-M111) |
| Xylan-1/XG | [CCRC-M108](http://glycomics.ccrc.uga.edu/wall2/jsp/abdetails.jsp?abnumber=149&amp;abname=CCRC-M108) |
|  | [CCRC-M109](http://glycomics.ccrc.uga.edu/wall2/jsp/abdetails.jsp?abnumber=150&amp;abname=CCRC-M109) |
|  |  |
|  | [CCRC-M119](http://glycomics.ccrc.uga.edu/wall2/jsp/abdetails.jsp?abnumber=106&amp;abname=CCRC-M119) |
| Xylan-2 | [CCRC-M115](http://glycomics.ccrc.uga.edu/wall2/jsp/abdetails.jsp?abnumber=110&amp;abname=CCRC-M115) |
|  | [CCRC-M110](http://glycomics.ccrc.uga.edu/wall2/jsp/abdetails.jsp?abnumber=167&amp;abname=CCRC-M110) |
|  | [CCRC-M105](http://glycomics.ccrc.uga.edu/wall2/jsp/abdetails.jsp?abnumber=165&amp;abname=CCRC-M105) |
|  |  |
|  | [CCRC-M117](http://glycomics.ccrc.uga.edu/wall2/jsp/abdetails.jsp?abnumber=108&amp;abname=CCRC-M117) |
|  | [CCRC-M113](http://glycomics.ccrc.uga.edu/wall2/jsp/abdetails.jsp?abnumber=171&amp;abname=CCRC-M113) |
| Xylan-3 | [CCRC-M120](http://glycomics.ccrc.uga.edu/wall2/jsp/abdetails.jsp?abnumber=105&amp;abname=CCRC-M120) |
|  | [CCRC-M118](http://glycomics.ccrc.uga.edu/wall2/jsp/abdetails.jsp?abnumber=107&amp;abname=CCRC-M118) |
|  | [CCRC-M116](http://glycomics.ccrc.uga.edu/wall2/jsp/abdetails.jsp?abnumber=109&amp;abname=CCRC-M116) |
|  | [CCRC-M114](http://glycomics.ccrc.uga.edu/wall2/jsp/abdetails.jsp?abnumber=111&amp;abname=CCRC-M114) |
|  |  |
| Xylan-4 | CCRC-M154 |
|  | CCRC-M150 |

|  | CCRC-M144 |
| --- | --- |
| Xylan-5 | CCRC-M146 |
|  | CCRC-M145 |
|  | CCRC-M155 |
|  |  |
|  | CCRC-M153 |
|  | CCRC-M151 |
| Xylan-6 | CCRC-M148 |
|  | CCRC-M140 |
|  | CCRC-M139 |
|  | CCRC-M138 |
|  |  |
|  | CCRC-M160 |
| Xylan-7 | [CCRC-M137](http://glycomics.ccrc.uga.edu/wall2/jsp/abdetails.jsp?abnumber=173&amp;abname=CCRC-M137) |
|  | CCRC-M152 |
|  | CCRC-M149 |
|  |  |
|  | [CCRC-M75](http://glycomics.ccrc.uga.edu/wall2/jsp/abdetails.jsp?abnumber=133&amp;abname=CCRC-M75) |
| Galactomannan-1 | [CCRC-M70](http://glycomics.ccrc.uga.edu/wall2/jsp/abdetails.jsp?abnumber=61&amp;abname=CCRC-M70) |
|  | [CCRC-M74](http://glycomics.ccrc.uga.edu/wall2/jsp/abdetails.jsp?abnumber=134&amp;abname=CCRC-M74) |
|  |  |
|  | CCRC-M166 |
| Galactomannan-2 | CCRC-M168 |
|  | CCRC-M174 |
|  | CCRC-M175 |
|  |  |
| Acetylated Mannan | CCRC-M169 |
|  | CCRC-M170 |
|  |  |
| β-Glucan | [LAMP](http://glycomics.ccrc.uga.edu/wall2/jsp/abdetails.jsp?abnumber=47&amp;abname=LAMP2H12H7) |
|  | [BG1](http://glycomics.ccrc.uga.edu/wall2/jsp/abdetails.jsp?abnumber=48&amp;abname=BG1) |
|  |  |
|  | [CCRC-M131](http://glycomics.ccrc.uga.edu/wall2/jsp/abdetails.jsp?abnumber=181&amp;abname=CCRC-M131) |
| HG  Backbone-1 | [CCRC-M38](http://glycomics.ccrc.uga.edu/wall2/jsp/abdetails.jsp?abnumber=45&amp;abname=CCRC-M38) |
|  | [JIM5](http://glycomics.ccrc.uga.edu/wall2/jsp/abdetails.jsp?abnumber=14&amp;abname=JIM5) |

| HG  Backbone-2 | [JIM136](http://glycomics.ccrc.uga.edu/wall2/jsp/abdetails.jsp?abnumber=57&amp;abname=JIM136) |
| --- | --- |
|  | [JIM7](http://glycomics.ccrc.uga.edu/wall2/jsp/abdetails.jsp?abnumber=13&amp;abname=JIM7) |
|  |  |
|  | [CCRC-M69](http://glycomics.ccrc.uga.edu/wall2/jsp/abdetails.jsp?abnumber=172&amp;abname=CCRC-M69) |
|  | [CCRC-M35](http://glycomics.ccrc.uga.edu/wall2/jsp/abdetails.jsp?abnumber=66&amp;abname=CCRC-M35) |
| RG-I  Backbone | [CCRC-M36](http://glycomics.ccrc.uga.edu/wall2/jsp/abdetails.jsp?abnumber=37&amp;abname=CCRC-M36) |
|  | [CCRC-M14](http://glycomics.ccrc.uga.edu/wall2/jsp/abdetails.jsp?abnumber=67&amp;abname=CCRC-M14) |
|  | [CCRC-M129](http://glycomics.ccrc.uga.edu/wall2/jsp/abdetails.jsp?abnumber=104&amp;abname=CCRC-M129) |
|  | [CCRC-M72](http://glycomics.ccrc.uga.edu/wall2/jsp/abdetails.jsp?abnumber=135&amp;abname=CCRC-M72) |
|  |  |
|  | [JIM3](http://glycomics.ccrc.uga.edu/wall2/jsp/abdetails.jsp?abnumber=79&amp;abname=JIM1) |
| Linseed Mucilage RG-I | [CCRC-M40](http://glycomics.ccrc.uga.edu/wall2/jsp/abdetails.jsp?abnumber=83&amp;abname=CCRC-M40) |
|  | CCRC-M161 |
|  | CCRC-M164 |
|  |  |
| Physcomitrella Pectin | [CCRC-M98](http://glycomics.ccrc.uga.edu/wall2/jsp/abdetails.jsp?abnumber=115&amp;abname=CCRC-M98) |
|  | [CCRC-M94](http://glycomics.ccrc.uga.edu/wall2/jsp/abdetails.jsp?abnumber=118&amp;abname=CCRC-M94) |
|  |  |
| RG-Ia | [CCRC-M5](http://glycomics.ccrc.uga.edu/wall2/jsp/abdetails.jsp?abnumber=81&amp;abname=CCRC-M5) |
|  | [CCRC-M2](http://glycomics.ccrc.uga.edu/wall2/jsp/abdetails.jsp?abnumber=8&amp;abname=CCRC-M2) |
|  |  |
|  | [JIM137](http://glycomics.ccrc.uga.edu/wall2/jsp/abdetails.jsp?abnumber=58&amp;abname=JIM137) |
| RG-Ib | [JIM101](http://glycomics.ccrc.uga.edu/wall2/jsp/abdetails.jsp?abnumber=55&amp;abname=JIM101) |
|  | [CCRC-M61](http://glycomics.ccrc.uga.edu/wall2/jsp/abdetails.jsp?abnumber=138&amp;abname=CCRC-M61) |
|  | [CCRC-M30](http://glycomics.ccrc.uga.edu/wall2/jsp/abdetails.jsp?abnumber=33&amp;abname=CCRC-M30) |
|  |  |
|  | [CCRC-M23](http://glycomics.ccrc.uga.edu/wall2/jsp/abdetails.jsp?abnumber=92&amp;abname=CCRC-M23) |
|  | [CCRC-M17](http://glycomics.ccrc.uga.edu/wall2/jsp/abdetails.jsp?abnumber=74&amp;abname=CCRC-M17) |
| RG-Ic | [CCRC-M19](http://glycomics.ccrc.uga.edu/wall2/jsp/abdetails.jsp?abnumber=0&amp;abname=CCRC-M19) |
|  | [CCRC-M18](http://glycomics.ccrc.uga.edu/wall2/jsp/abdetails.jsp?abnumber=0&amp;abname=CCRC-M18) |
|  | [CCRC-M56](http://glycomics.ccrc.uga.edu/wall2/jsp/abdetails.jsp?abnumber=141&amp;abname=CCRC-M56) |
|  | [CCRC-M16](http://glycomics.ccrc.uga.edu/wall2/jsp/abdetails.jsp?abnumber=73&amp;abname=CCRC-M16) |
|  |  |
|  | [CCRC-M60](http://glycomics.ccrc.uga.edu/wall2/jsp/abdetails.jsp?abnumber=139&amp;abname=CCRC-M60) |
| RG-I/Arabinogalactan | [CCRC-M41](http://glycomics.ccrc.uga.edu/wall2/jsp/abdetails.jsp?abnumber=82&amp;abname=CCRC-M41) |
|  | [CCRC-M80](http://glycomics.ccrc.uga.edu/wall2/jsp/abdetails.jsp?abnumber=128&amp;abname=CCRC-M80) |
|  | [CCRC-M79](http://glycomics.ccrc.uga.edu/wall2/jsp/abdetails.jsp?abnumber=129&amp;abname=CCRC-M79) |

|  | [CCRC-M44](http://glycomics.ccrc.uga.edu/wall2/jsp/abdetails.jsp?abnumber=68&amp;abname=CCRC-M44) |
| --- | --- |
|  | [CCRC-M33](http://glycomics.ccrc.uga.edu/wall2/jsp/abdetails.jsp?abnumber=75&amp;abname=CCRC-M33) |
|  | [CCRC-M32](http://glycomics.ccrc.uga.edu/wall2/jsp/abdetails.jsp?abnumber=35&amp;abname=CCRC-M32) |
|  | [CCRC-M13](http://glycomics.ccrc.uga.edu/wall2/jsp/abdetails.jsp?abnumber=43&amp;abname=CCRC-M13) |
|  | [CCRC-M42](http://glycomics.ccrc.uga.edu/wall2/jsp/abdetails.jsp?abnumber=86&amp;abname=CCRC-M42) |
|  | [CCRC-M24](http://glycomics.ccrc.uga.edu/wall2/jsp/abdetails.jsp?abnumber=93&amp;abname=CCRC-M24) |
|  | [CCRC-M12](http://glycomics.ccrc.uga.edu/wall2/jsp/abdetails.jsp?abnumber=71&amp;abname=CCRC-M12) |
|  | [CCRC-M7](http://glycomics.ccrc.uga.edu/wall2/jsp/abdetails.jsp?abnumber=3&amp;abname=CCRC-M7) |
|  | [CCRC-M77](http://glycomics.ccrc.uga.edu/wall2/jsp/abdetails.jsp?abnumber=131&amp;abname=CCRC-M77) |
|  | [CCRC-M25](http://glycomics.ccrc.uga.edu/wall2/jsp/abdetails.jsp?abnumber=84&amp;abname=CCRC-M25) |
|  | [CCRC-M9](http://glycomics.ccrc.uga.edu/wall2/jsp/abdetails.jsp?abnumber=69&amp;abname=CCRC-M9) |
|  | [CCRC-M128](http://glycomics.ccrc.uga.edu/wall2/jsp/abdetails.jsp?abnumber=183&amp;abname=CCRC-M128) |
|  | [CCRC-M126](http://glycomics.ccrc.uga.edu/wall2/jsp/abdetails.jsp?abnumber=184&amp;abname=CCRC-M126) |
|  | [CCRC-M134](http://glycomics.ccrc.uga.edu/wall2/jsp/abdetails.jsp?abnumber=102&amp;abname=CCRC-M134) |
|  | [CCRC-M125](http://glycomics.ccrc.uga.edu/wall2/jsp/abdetails.jsp?abnumber=185&amp;abname=CCRC-M125) |
|  | [CCRC-M123](http://glycomics.ccrc.uga.edu/wall2/jsp/abdetails.jsp?abnumber=187&amp;abname=CCRC-M123) |
|  | [CCRC-M122](http://glycomics.ccrc.uga.edu/wall2/jsp/abdetails.jsp?abnumber=188&amp;abname=CCRC-M122) |
|  | [CCRC-M121](http://glycomics.ccrc.uga.edu/wall2/jsp/abdetails.jsp?abnumber=189&amp;abname=CCRC-M121) |
|  | [CCRC-M112](http://glycomics.ccrc.uga.edu/wall2/jsp/abdetails.jsp?abnumber=169&amp;abname=CCRC-M112) |
|  | [CCRC-M21](http://glycomics.ccrc.uga.edu/wall2/jsp/abdetails.jsp?abnumber=88&amp;abname=CCRC-M21) |
|  | [JIM131](http://glycomics.ccrc.uga.edu/wall2/jsp/abdetails.jsp?abnumber=94&amp;abname=JIM131) |
|  | [CCRC-M22](http://glycomics.ccrc.uga.edu/wall2/jsp/abdetails.jsp?abnumber=46&amp;abname=CCRC-M22) |
|  | [JIM132](http://glycomics.ccrc.uga.edu/wall2/jsp/abdetails.jsp?abnumber=56&amp;abname=JIM132) |
|  | [JIM1](http://glycomics.ccrc.uga.edu/wall2/jsp/abdetails.jsp?abnumber=79&amp;abname=JIM1) |
|  | [CCRC-M15](http://glycomics.ccrc.uga.edu/wall2/jsp/abdetails.jsp?abnumber=72&amp;abname=CCRC-M15) |
|  | [CCRC-M8](http://glycomics.ccrc.uga.edu/wall2/jsp/abdetails.jsp?abnumber=29&amp;abname=CCRC-M8) |
|  | [JIM16](http://glycomics.ccrc.uga.edu/wall2/jsp/abdetails.jsp?abnumber=62&amp;abname=JIM16) |
|  |  |
|  | [JIM93](http://glycomics.ccrc.uga.edu/wall2/jsp/abdetails.jsp?abnumber=117&amp;abname=JIM93) |
|  | [JIM94](http://glycomics.ccrc.uga.edu/wall2/jsp/abdetails.jsp?abnumber=95&amp;abname=JIM94) |
| Arabinogalactan-1 | [JIM11](http://glycomics.ccrc.uga.edu/wall2/jsp/abdetails.jsp?abnumber=41&amp;abname=JIM11) |
|  | [MAC204](http://glycomics.ccrc.uga.edu/wall2/jsp/abdetails.jsp?abnumber=23&amp;abname=MAC204) |
|  | [JIM20](http://glycomics.ccrc.uga.edu/wall2/jsp/abdetails.jsp?abnumber=91&amp;abname=JIM20) |

|  | [JIM14](http://glycomics.ccrc.uga.edu/wall2/jsp/abdetails.jsp?abnumber=31&amp;abname=JIM14) |
| --- | --- |
|  | [JIM19](http://glycomics.ccrc.uga.edu/wall2/jsp/abdetails.jsp?abnumber=44&amp;abname=JIM19) |
| Arabinogalactan-2 | [JIM12](http://glycomics.ccrc.uga.edu/wall2/jsp/abdetails.jsp?abnumber=191&amp;abname=JIM12) |
|  | [CCRC-M133](http://glycomics.ccrc.uga.edu/wall2/jsp/abdetails.jsp?abname=CCRC-M133) |
|  | [CCRC-M107](http://glycomics.ccrc.uga.edu/wall2/jsp/abdetails.jsp?abnumber=166&amp;abname=CCRC-M107) |
|  |  |
|  | [JIM4](http://glycomics.ccrc.uga.edu/wall2/jsp/abdetails.jsp?abnumber=40&amp;abname=JIM4) |
|  | [CCRC-M31](http://glycomics.ccrc.uga.edu/wall2/jsp/abdetails.jsp?abnumber=34&amp;abname=CCRC-M31) |
|  | [JIM17](http://glycomics.ccrc.uga.edu/wall2/jsp/abdetails.jsp?abnumber=39&amp;abname=JIM17) |
|  | [CCRC-M26](http://glycomics.ccrc.uga.edu/wall2/jsp/abdetails.jsp?abnumber=85&amp;abname=CCRC-M26) |
| Arabinogalactan-3 | [JIM15](http://glycomics.ccrc.uga.edu/wall2/jsp/abdetails.jsp?abnumber=32&amp;abname=JIM15) |
|  | [JIM8](http://glycomics.ccrc.uga.edu/wall2/jsp/abdetails.jsp?abnumber=80&amp;abname=JIM8) |
|  | [CCRC-M85](http://glycomics.ccrc.uga.edu/wall2/jsp/abdetails.jsp?abnumber=121&amp;abname=CCRC-M85) |
|  | [CCRC-M81](http://glycomics.ccrc.uga.edu/wall2/jsp/abdetails.jsp?abnumber=127&amp;abname=CCRC-M81) |
|  | [MAC266](http://glycomics.ccrc.uga.edu/wall2/jsp/abdetails.jsp?abnumber=98&amp;abname=MAC266) |
|  | [PN 16.4B4](http://glycomics.ccrc.uga.edu/wall2/jsp/abdetails.jsp?abnumber=11&amp;abname=PN%2016.4B4) |
|  |  |
|  | [MAC207](http://glycomics.ccrc.uga.edu/wall2/jsp/abdetails.jsp?abnumber=22&amp;abname=MAC207) |
|  | [JIM133](http://glycomics.ccrc.uga.edu/wall2/jsp/abdetails.jsp?abnumber=96&amp;abname=JIM133) |
| Arabinogalactan-4 | [JIM13](http://glycomics.ccrc.uga.edu/wall2/jsp/abdetails.jsp?abnumber=30&amp;abname=JIM13) |
|  | [CCRC-M92](http://glycomics.ccrc.uga.edu/wall2/jsp/abdetails.jsp?abnumber=119&amp;abname=CCRC-M92) |
|  | [CCRC-M91](http://glycomics.ccrc.uga.edu/wall2/jsp/abdetails.jsp?abnumber=120&amp;abname=CCRC-M91) |
|  | [CCRC-M78](http://glycomics.ccrc.uga.edu/wall2/jsp/abdetails.jsp?abnumber=130&amp;abname=CCRC-M78) |
|  |  |
| Unidentified | [MAC265](http://glycomics.ccrc.uga.edu/wall2/jsp/abdetails.jsp?abnumber=97&amp;abname=MAC265) |
|  | [CCRC-M97](http://glycomics.ccrc.uga.edu/wall2/jsp/abdetails.jsp?abnumber=116&amp;abname=CCRC-M97) |
